# Supplementary material for: Global Terrapin Character-Based DNA Barcodes: Assessment of the Mitochondrial COI Gene and Conservation Status Revealed a Putative Cryptic Species
Source: Animals (Basel). 2023 May 23;13(11):1720. doi: 10.3390/ani13111720 (PMC10251852; doi:10.3390/ani13111720)
Supplement: Supplementary file 1 [file animals-13-01720-s001.zip › animals-2195225-supplementary.pdf]

Supplementary Figures

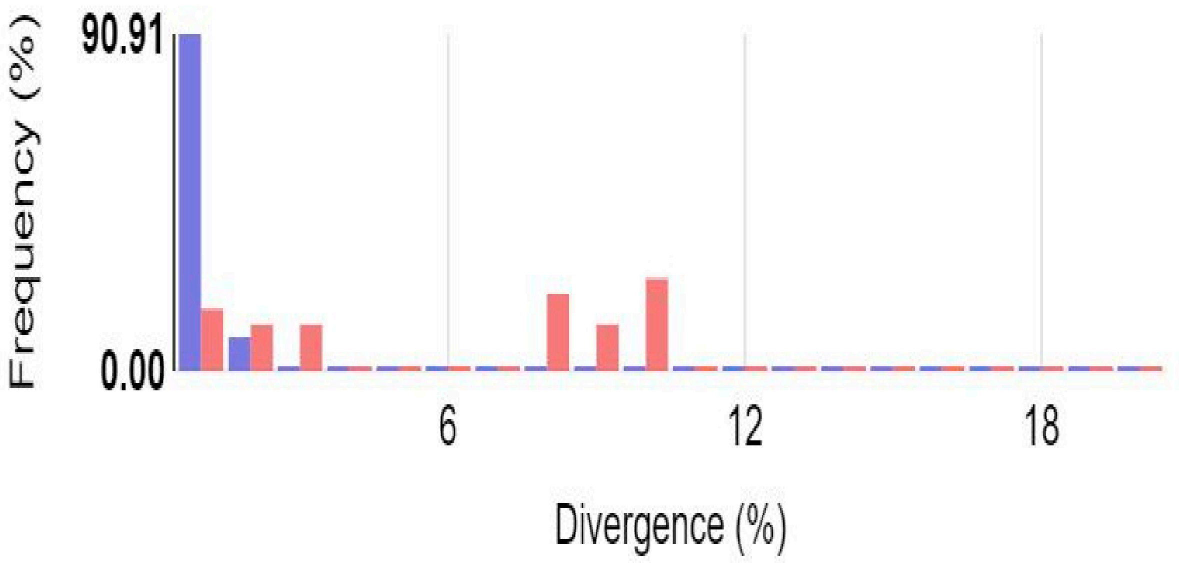

Supplement Figure S1. The within-species distribution is normalised to reduce bias in sampling at the species level. This distribution is shown in the histogram compares the distribution of normalised divergences between species (blue) and genera (red).

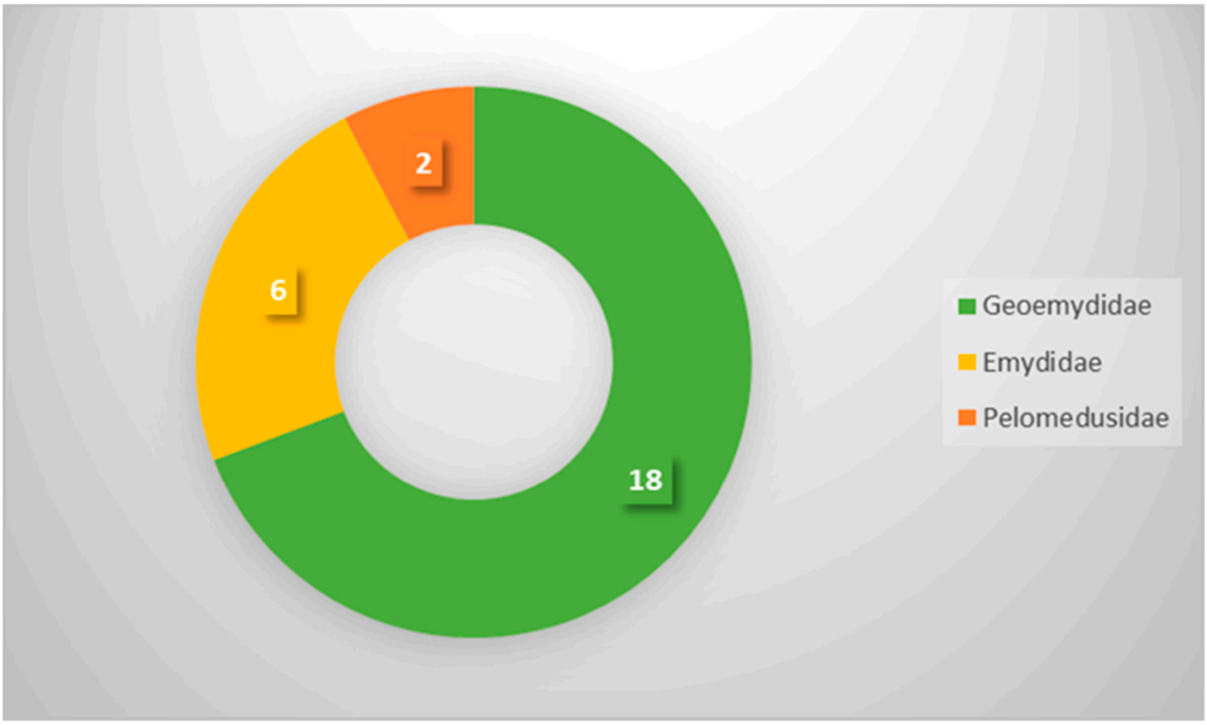

Supplement Figure S2. The family of sampled terrapins.

Supplementary Table

Supplement Table S1. Terrapin COI sequence composition (from 26 samples).

Sequence composition (%)	Min	Mean	Max	SE
Guanine	14.15	16.88	18.66	0.2114
Citocyne	23.96	27.21	30.34	0.4152
Adenine	25	27.5	30.31	0.3073
Tyrosine	26	28.41	30.61	0.2823
Guanine-Citocyne	40.16	44.09	47.71	0.5384
Guanine-Citocyne codon postion 1	48.54	52.62	55.09	0.3597
Guanine-Citocyne codon postion 2	40.86	43.21	45.04	0.2275
Guanine-Citocyne codon postion 3	26.27	36.46	45.16	1.2697

Supplement Table S2. DNA barcodes for terrapin based on pure diagnostic characters at selected nucleotide positions. The transition site was highlighted in yellow.

Species (n)	COI variable sites																																																
																																			1	1	1	1	1	1	1	1	1	1	1	1	1	1	1
						1	1	1	2	2	2	2	3	3	4	4	4	5	5	5	5	6	6	6	6	7	7	7	7	8	8	8	8	8	9	9	0	0	0	1	1	2	2	2	2	3	3	3	4	4	4
	3	4	6	9	2	3	5	1	4	5	7	3	9	2	5	8	1	2	4	7	0	3	6	9	2	5	8	9	1	2	4	7	8	6	9	2	5	8	1	7	0	3	4	9	2	5	8	1	4	7
Batagur baska (2)	T	A	T	T	A	G	C	C	C	G	C	T	C	A	T	G	G	A	C	A	T	A	A	C	A	C	A	C	T	G	C	A	C	C	A	C	G	C	A	C	C	T	A	C	C	A	C	C	A	T
Batagur borneoensis (2)	C	.	C	C	.	.	.	.	.	.	.	.	T	.	G	.	A	.	.	G	.	C	C	.	.	.	.	.	.	.	.	C	.	.	G	.	.	.	.	.	.	.	.	.	T	.	.	.	.	.
Morenia ocellata (2)	A	.	C	A	.	.	T	.	.	.	T	.	T	.	.	A	A	.	.	.	C	C	C	.	.	.	.	.	.	A	T	.	.	T	.	A	T	.	.	.	T	.	.	.	.	.	.	.	.	.
Malaclemys terrapin (2)	.	.	C	C	.	.	.	T	T	A	T	.	.	G	.	A	A	.	T	.	.	T	G	T	.	T	.	.	.	.	.	C	T	T	.	A	A	T	G	T	A	.	.	.	T	.	T	T	.	.
Emys orbicularis (2)	.	.	C	.	.	.	.	.	.	A	T	C	.	G	C	A	A	G	T	.	.	T	.	T	.	T	.	.	.	.	A	.	T	.	.	A	A	T	.	T	A	.	.	T	T	.	T	.	.	.
Melanochelys trijuga (2)	C	.	C	.	C	.	.	T	T	A	.	.	.	.	.	A	T	.	.	.	.	C	C	T	T	.	.	.	.	T	A	C	T	T	.	.	A	.	G	.	.	C	.	.	.	.	.	.	.	.
Rhinoclemmys rubida (1)	.	.	C	C	.	.	.	T	T	A	T	.	.	.	.	.	A	.	T	.	.	.	C	.	.	T	.	.	.	.	A	.	T	.	.	G	A	T	.	T	A	.	.	T	T	.	T	.	.	C
Trachemys scripta elegans (2)	.	G	C	C	.	.	.	T	.	A	T	.	.	.	.	A	A	.	T	.	.	C	G	T	G	.	.	.	C	.	G	.	T	T	.	G	A	.	G	T	A	.	.	.	T	.	T	T	G	.
Pelusios sinuatus (2)	C	.	.	C	.	T	.	T	T	A	T	.	T	.	.	A	T	.	T	.	.	.	.	.	.	.	.	T	G	.	.	C	.	T	.	T	A	T	.	.	A	C	C	.	.	.	.	.	.	A
Siebenrockiella crassicollis (2)	C	.	C	.	.	.	.	.	.	.	.	.	.	.	C	A	A	.	T	.	C	G	.	.	.	.	G	.	.	.	T	C	.	T	.	.	A	.	G	.	G	A	.	T	T	G	.	T	.	A
Mauremys caspica (2)	.	.	C	.	.	.	.	.	T	A	.	.	.	G	.	A	T	G	T	.	C	T	C	T	.	.	.	.	.	.	A	C	T	.	G	G	A	T	G	.	A	.	.	.	T	.	.	.	.	.
Batagur affinis edwardmolli (3)	.	.	.	.	.	.	.	.	.	.	.	.	.	.	.	.	.	.	.	.	.	.	.	.	.	.	.	.	.	.	.	.	.	.	.	.	A	.	.	.	.	.	.	.	.	.	.	.	G	.

Species (n)	1	1	1	1	1	1	1	1	1	1	1	1	1	1	1	1	1	1	1	1	1	1	1	1	1	1	1	1	2	2	2	2	2	2	2	2	2	2	2	2	2	2	2	2	2	2	2	2	2	2
	4	5	5	5	5	6	6	6	6	6	6	6	7	7	7	7	8	8	8	8	8	8	8	9	9	9	9	9	0	0	0	1	1	1	2	2	2	2	3	3	3	4	4	4	4	4	5	5	5	6
	8	0	3	6	9	0	2	3	5	6	8	9	1	2	4	7	0	3	4	5	6	7	9	2	3	5	8	9	1	4	7	0	6	9	2	5	8	9	2	4	7	0	1	3	6	9	2	5	8	1
Batagur baska (2)	C	G	G	A	C	C	A	C	C	T	A	C	A	C	A	C	A	C	G	G	A	G	T	A	T	G	T	G	G	C	A	A	T	G	C	A	T	T	G	C	A	C	T	A	C	T	T	T	C	C
Batagur borneoensis (2)	.	.	A	.	.	.	.	.	.	C	.	.	.	.	.	.	.	T	.	.	.	.	.	.	.	A	.	.	C	.	.	G	.	A	.	.	.	.	.	.	.	T	.	G	.	.	C	.	.	.
Morenia ocellata (2)	T	A	A	.	.	.	.	.	.	C	T	.	.	.	.	.	.	A	.	.	G	.	A	.	.	A	C	.	A	.	T	.	.	A	.	.	A	C	.	T	.	.	.	.	.	.	C	.	.	T
Malaclemys terrapin (2)	T	A	C	T	A	T	.	T	A	.	.	.	.	.	.	A	.	A	.	.	.	A	.	.	G	A	G	.	A	.	.	C	.	A	T	C	A	.	.	.	.	.	C	.	.	C	C	.	.	T
Emys orbicularis (2)	T	A	T	.	.	.	.	.	A	C	.	.	T	.	.	A	.	A	.	.	.	A	.	.	G	A	G	.	A	.	.	T	.	A	.	C	G	C	.	.	.	.	.	.	T	.	C	.	.	T
Melanochelys trijuga (2)	.	C	C	.	A	.	.	.	T	C	.	.	C	.	.	.	.	A	.	.	.	A	.	.	G	A	C	.	A	T	T	T	A	A	.	G	A	C	.	T	.	.	C	.	.	C	C	.	.	T
Rhinoclemmys rubida (1)	.	.	T	T	T	.	T	.	T	C	.	T	.	T	.	.	.	A	.	.	.	A	.	G	G	A	C	.	A	.	.	T	C	A	.	C	.	C	.	.	.	T	.	G	.	C	.	C	.	T
Trachemys scripta elegans (2)	T	A	C	T	A	T	.	T	A	.	.	.	C	.	.	A	.	A	.	.	.	A	.	.	G	A	C	.	A	.	.	C	.	A	.	C	A	.	.	.	.	T	.	.	.	C	C	.	.	T
Pelusios sinuatus (2)	T	A	T	.	A	T	.	.	A	C	.	A	.	.	C	.	.	.	A	T	.	A	.	.	G	A	A	.	T	A	.	C	A	T	.	T	A	C	T	A	T	T	C	.	.	.	C	.	A	T
Siebenrockiella crassicollis (2)	T	A	A	.	.	T	.	.	.	C	T	.	T	.	.	.	.	T	.	.	T	A	.	G	G	A	C	.	A	A	.	.	.	A	.	.	A	.	.	.	.	T	C	C	.	C	C	.	.	T
Mauremys caspica (2)	.	A	A	G	.	.	.	.	T	.	.	.	T	.	G	.	C	A	.	.	.	.	.	.	G	A	C	.	A	.	.	C	.	.	T	.	A	.	.	T	.	.	C	.	.	C	.	C	.	T
Batagur affinis edwardmolli (3)	.	.	A	.	.	.	.	.	.	.	.	.	.	.	.	.	.	.	.	.	.	.	.	.	.	.	.	.	.	T	.	.	C	.	.	.	.	.	.	.	.	.	.	.	.	.	.	.	.	.

Species (n)	2	2	2	2	2	2	2	2	2	2	2	2	2	2	3	3	3	3	3	3	3	3	3	3	3	3	3	3	3	3	3	3	3	3	3	3	3	3	3	3	3	3	3	3	3	3	3	3	3	3
	6	6	7	7	7	7	8	8	8	8	9	9	9	9	0	0	0	0	1	1	1	1	1	2	2	2	3	3	3	3	4	4	4	5	5	5	5	5	5	6	6	6	6	6	6	6	6	7	7	7
	7	8	0	1	6	9	2	5	8	9	1	4	7	9	0	3	6	9	0	2	5	8	9	1	4	7	0	3	6	8	2	5	8	1	2	4	7	8	9	0	3	4	5	6	7	8	9	0	1	2
Batagur baska (2)	C	C	A	A	T	C	C	C	C	C	G	C	A	T	A	C	A	C	C	G	G	C	A	T	C	T	C	C	A	C	C	C	A	A	T	T	A	G	C	C	A	A	C	C	C	A	G	T	A	C
Batagur borneoensis (2)	.	.	.	.	.	.	.	.	.	.	.	T	.	.	.	.	.	.	.	A	.	.	.	C	T	.	.	.	.	.	.	.	.	.	.	C	.	.	.	.	.	.	.	.	.	.	A	.	.	.
Morenia ocellata (2)	.	.	.	.	C	.	A	T	.	.	A	.	T	.	.	.	.	T	T	A	.	.	.	C	.	.	.	.	.	.	T	.	.	.	C	C	.	.	.	A	.	.	.	A	.	.	A	.	.	.
Malaclemys terrapin (2)	.	.	.	.	C	T	T	T	.	T	A	A	.	.	.	.	.	T	.	A	.	T	.	.	.	C	T	.	.	.	T	.	.	.	.	C	.	.	.	T	.	T	.	A	.	.	A	.	.	.
Emys orbicularis (2)	.	.	.	.	.	T	T	.	.	T	A	T	T	.	.	T	.	T	T	A	.	T	.	C	T	.	T	.	.	.	T	.	.	.	.	C	.	.	.	.	.	T	.	A	.	.	A	.	.	.
Melanochelys trijuga (2)	T	T	G	.	.	.	.	T	.	T	A	T	T	.	.	A	.	T	T	A	C	T	.	.	.	.	.	T	T	.	T	.	G	G	.	C	C	.	.	T	.	T	.	A	.	.	A	.	.	.
Rhinoclemmys rubida (1)	T	T	.	.	.	T	.	T	.	.	A	T	T	.	G	A	C	T	.	A	.	T	.	C	.	C	T	T	.	.	.	T	.	.	.	C	.	.	.	.	.	.	.	.	.	.	A	.	.	T
Trachemys scripta elegans (2)	.	.	G	.	C	T	T	T	T	T	A	A	.	.	.	T	.	T	.	A	A	T	.	.	.	C	T	.	.	.	T	.	.	.	.	C	.	.	.	.	G	T	.	A	.	.	A	.	.	.
Pelusios sinuatus (2)	T	.	.	G	.	.	A	A	.	.	A	A	.	C	.	.	.	.	.	A	A	.	C	C	.	C	.	T	T	T	T	.	.	.	A	C	.	A	A	.	.	.	A	.	T	T	C	C	T	A
Siebenrockiella crassicollis (2)	T	.	.	.	.	.	.	.	.	.	C	.	T	.	.	A	.	.	T	.	.	T	.	C	.	.	.	T	.	.	A	T	.	.	C	.	C	.	.	.	.	T	.	A	.	.	A	.	.	.
Mauremys caspica (2)	T	.	.	.	C	T	.	.	.	.	A	.	T	.	.	A	.	T	T	A	.	.	.	C	.	.	.	.	.	.	T	.	.	.	.	.	.	.	.	T	.	T	.	A	.	.	A	.	.	.
Batagur affinis edwardmolli (3)	.	.	.	.	.	.	.	.	.	.	.	.	.	.	.	.	.	.	.	A	.	.	.	.	.	.	.	.	.	.	.	.	.	.	.	.	.	.	.	.	.	.	.	.	.	.	A	.	.	.

Species (n)	3	3	3	3	3	3	3	3	3	3	3	4	4	4	4	4	4	4	4	4	4	4	4	4	4	4	4	4	4	4	4	4	4	4	4	4	4	4	4	4	4	4	4	4	4	4	4	4	5
	7	7	7	8	8	8	8	9	9	9	9	0	0	0	0	1	1	1	1	1	2	2	2	2	2	3	3	3	3	4	4	5	5	5	5	6	6	6	6	7	7	7	8	8	8	9	9	9	0
	3	5	7	1	2	4	7	0	3	6	9	0	2	5	8	1	2	5	7	8	1	3	4	6	9	0	2	5	8	1	4	0	3	6	9	0	2	3	5	1	7	8	0	3	9	2	5	8	1
Batagur baska (2)	C	A	C	C	T	A	T	G	A	T	A	C	T	T	A	C	G	C	G	C	C	G	C	T	A	C	C	A	A	T	T	A	C	T	A	C	A	C	C	T	C	C	G	T	C	C	T	C	T
Batagur borneoensis (2)	.	.	.	T	.	.	.	A	.	.	G	.	.	.	.	.	.	.	A	.	.	.	.	C	.	.	T	.	.	.	.	.	.	C	.	.	.	.	T	.	.	.	A	.	.	.	.	T	C
Morenia ocellata (2)	.	.	.	T	.	.	.	T	.	A	.	.	A	.	C	.	.	.	A	T	T	A	.	.	.	T	A	.	T	A	.	.	.	.	.	.	.	.	T	.	.	.	A	.	.	T	.	.	A
Malaclemys terrapin (2)	.	.	.	.	C	.	.	.	.	A	.	.	.	.	.	T	.	.	A	T	.	A	.	A	G	.	A	.	.	A	.	C	.	.	.	.	.	T	A	.	.	.	A	.	.	.	.	.	.
Emys orbicularis (2)	.	.	.	.	C	G	.	A	.	A	.	.	.	.	C	T	.	.	A	T	.	A	T	A	.	T	A	.	.	A	.	C	.	.	.	.	.	.	T	C	.	T	A	.	.	.	.	.	.
Melanochelys trijuga (2)	.	.	.	.	.	.	.	A	G	A	.	.	C	C	.	.	A	.	.	.	.	A	.	C	.	.	G	.	.	.	C	T	.	.	.	.	.	.	T	.	.	.	A	.	.	.	C	T	.
Rhinoclemmys rubida (1)	.	.	.	A	.	.	.	.	.	C	.	.	C	C	.	.	.	.	A	.	.	.	.	C	.	.	G	C	.	.	.	C	.	.	.	T	.	.	A	.	T	.	A	C	.	.	.	.	.
Trachemys scripta elegans (2)	.	.	.	.	C	.	.	.	.	A	.	.	.	.	.	T	.	.	A	T	T	A	.	A	G	.	A	.	C	A	.	C	.	.	.	.	.	T	A	C	.	.	A	.	T	.	.	.	.
Pelusios sinuatus (2)	G	C	T	A	C	.	.	T	.	A	.	T	A	C	.	.	A	T	A	.	.	C	T	A	C	.	A	.	.	.	A	C	T	.	.	.	T	T	A	.	.	.	A	C	T	.	C	T	C
Siebenrockiella crassicollis (2)	.	.	.	T	C	.	C	.	.	A	.	.	.	.	G	.	.	.	A	.	.	.	.	C	.	T	A	T	C	C	.	G	.	C	G	.	.	.	G	C	T	.	A	C	.	T	.	.	G
Mauremys caspica (2)	.	.	.	T	.	.	.	A	.	A	.	.	.	C	.	.	.	.	A	T	T	A	.	.	.	.	.	.	.	.	C	T	T	.	.	.	.	.	.	C	.	.	A	.	.	.	C	.	.
Batagur affinis edwardmolli (3)	.	G	.	.	.	.	.	.	.	.	.	.	.	.	.	.	.	.	.	.	.	.	.	.	.	.	.	.	.	C	.	.	.	.	.	.	.	.	.	.	.	.	.	.	.	.	.	.	.

Supplement Table S3. The number of terrapin sequences, species, genera, and families is analysed in the present study.

Family	Species	Genus	Sequences
Geoemydidae	8	6	18
Emydidae	3	3	6
Pelomedusidae	1	1	2
